# The value of metabolic imaging to predict tumour response after chemoradiation in locally advanced rectal cancer

**DOI:** 10.1186/1748-717X-5-119

**Published:** 2010-12-15

**Authors:** Pablo Palma, Raquel Conde-Muíño, Antonio Rodríguez-Fernández, Inmaculada Segura-Jiménez, Rocío Sánchez-Sánchez, Javier Martín-Cano, Manuel Gómez-Río, José A Ferrón, José M Llamas-Elvira

**Affiliations:** 1 Division of Colon &Rectal Surgery - Department of Surgery, HUVN Granada - Spain; 2 Department of Nuclear Medicine, HUVN Granada - Spain

## Abstract

**Background:**

We aim to investigate the possibility of using 18F-positron emission tomography/computer tomography (PET-CT) to predict the histopathologic response in locally advanced rectal cancer (LARC) treated with preoperative chemoradiation (CRT).

**Methods:**

The study included 50 patients with LARC treated with preoperative CRT. All patients were evaluated by PET-CT before and after CRT, and results were compared to histopathologic response quantified by tumour regression grade (patients with TRG 1-2 being defined as responders and patients with grade 3-5 as non-responders). Furthermore, the predictive value of metabolic imaging for pathologic complete response (ypCR) was investigated.

**Results:**

Responders and non-responders showed statistically significant differences according to Mandard's criteria for maximum standardized uptake value (SUV_max_) before and after CRT with a specificity of 76,6% and a positive predictive value of 66,7%. Furthermore, SUV_max _values after CRT were able to differentiate patients with ypCR with a sensitivity of 63% and a specificity of 74,4% (positive predictive value 41,2% and negative predictive value 87,9%); This rather low sensitivity and specificity determined that PET-CT was only able to distinguish 7 cases of ypCR from a total of 11 patients.

**Conclusions:**

We conclude that 18-F PET-CT performed five to seven weeks after the end of CRT can visualise functional tumour response in LARC. In contrast, metabolic imaging with 18-F PET-CT is not able to predict patients with ypCR accurately.

## Background

Over the past decade neoadjuvant chemo-radiotherapy (CRT) has been increasingly employed in the treatment of locally advanced rectal cancer (LARC). Clinical trials have shown a reduction in tumour size and stage, as well as a significant reduced risk of local recurrence. Tumour responses to CRT, however, vary considerably. While pathological complete response is noted in up to 30 percent of patients who undergo preoperative CRT and evidence suggests that complete response is associated with better oncologic outcomes, serious side effects and even no response - after weeks of treatment -is observed in the remaining amount of patients [1].

The surgical approach largely depends on a valid assessment of the preoperative extent of the tumour, particularly for distally located tumours or those that have been assessed as being nonresectable during primary staging. Regarding the further treatment, some institutions raised the question whether radical surgery should be necessary for patients with clinical complete response to CRT [2,3]. Therefore, for the clinical practice, radiological prediction of the histopathological tumour response is quite attractive because it could enable response-guided modifications of the treatment protocol. Clinical assessment after CRT is known to be quite poor and conventional imaging modalities cannot distinguish fibrosis or scar from viable tumour cells in residual masses [4].

As a result, great demands are placed on imaging modalities that provide a combination of metabolic and morphologic information. Incorporation of 2-deoxy-2-[18F]fluoro-D-glucose (18-FDG) positron emission tomography (PET) scans in the management of patients with cancer has increased with the introduction of this modality into clinical practice [5].

After our preliminary experience with this technique dealing with staging of colorectal cancer [6], in the current prospective study we aim to specifically determine whether PET-CT scans could predict histopathological response in patients with LARC after treatment with preoperative CRT.

## Methods

### Patients characteristics

**Figure 1:**
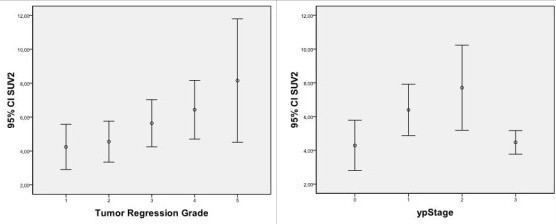
**SUV2 values classified by tumour regression grade criteria and ypStage criteria**. Points are mean values; error bars are 95% confidence interval.

**Figure 2:**
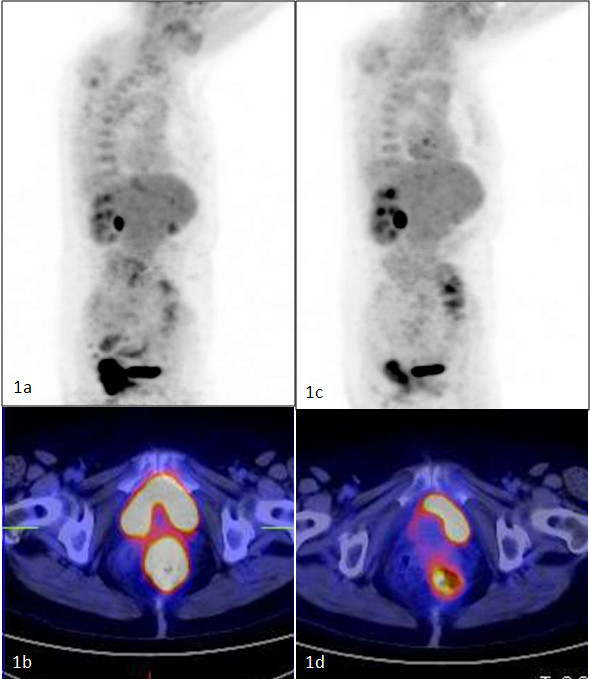
**Partial metabolic response to CRT**. 2A: Pre-CRT study - Intense rectal FDG uptake. 2B: Pre-CRT study - axial PET-CT images showing hypermetabolic rectal mass. 2C: Post-CRT study - Tumour volume is reduced but considerable tumour uptake is still present. 2D: Axial PET-CT images showing rectal mass in CT images with FDG uptake in PET images.

A cohort of 50 patients diagnosed with nonmetastasized LARC was included in this study (UICC Stage II and III). Preoperative TN staging was evaluated with magnetic resonance scan (MRI) and endorectal ultrasound (US). All patients received neoadjuvant radiotherapy (28 fractions of 1.8 Gy, 5 fractions/week) with concomitant chemotherapy (capecitabine, 825 mg/m2, twice daily alone or in combination with oxaliplatine 50 mg/m2 once weekly), followed by surgery 8 weeks after completion of CRT. All patients underwent sequential FDG-PET-CT imaging at two different time points: once prior to neoadjuvant therapy and once just prior to surgery (Figure 1 and 2).

### PET-CT imaging and processing

All PET-CT scans were performed by use of a dedicated Siemens Biograph 16, (Knoxville, Tennessee) with an axial field of view of 16.2 cm, a slice thickness of 3 mm, and a pixel spacing of 5.4 mm in both directions. The scanner is equipped with ultrafast detector electronics (Pico3D) and has a spatial resolution of approximately 6 mm at full-width at-half-maximum. PET imaging was done in three dimensions, requiring a proper scatter correction. CT-based attenuation correction was performed. PET images were reconstructed from the acquired list mode data, using Fourier rebinding and ordered subset expectation maximization reconstruction (three dimensional) with two iterations and eight subsets (*Ordered subset expectation maximization*). After a fasting period of at least 6 hours prior to FDG injection, patients received an intravenous injection of 18-FDG, with the activity normalized for the weight of the patient, followed by an injection of physiologic saline (10 ml). After an uptake period of 60 minutes, the patient was positioned on a flat tabletop, using a movable laser alignment system in a ''head-first supine'' position with the arm elevated over the head to avoid beam hardening artefacts as well as errors caused by truncation of the field of view. A PET-CT scan of the whole body was performed using an acquisition time of 2 to 4 (depending of the patient's weight) minutes per bed position. Additionally, all PET data were normalized for the blood glucose level measured shortly before 18-FDG administration (Glucocard G meter; Menarini Diagnostic, Florence).

### PET analysis

Standardized uptake values (SUV) were calculated for each tumour (Syngo Multimodality Workplace vs2009A; Siemens Medical Solutions; Siemens AG, Berlin). SUV is a measurement of the uptake in a tumour normalized on the basis of a distribution volume. It is calculated as follows:

$${\text{SUV}}_{glu}=\left[{\text{Act}}_{voi}\left(\text{kBq}/\text{ml}\right)/{\text{Act}}_{\text{administered}}\left(\text{MBq}\right)/\text{BW}\left(\text{Kg}\right)]\text{×}[{\text{Gluc}}_{\text{plasma}}\left(\text{mmol}/\text{L}\right)/\text{5}.0\left(\text{mmol}/\text{L}\right)\right]$$

In these calculations, Act_voi _is the activity measured in the volume of interest (this is equals the voxel with highest uptake in tumour), Act_administered _is the administered activity corrected for the physical decay of FDG to the start of acquisition, and BW is body weight. Dedicated software was used to calculate the SUV_max _within the tumour before (SUV1) and after CRT (SUV2). Subsequently, the regression index (RI), indicating the percent reduction relative to the pre-treatment measured value, were calculated (RI = [(SUV1-SUV2)/SUV1] × 100) and correlated to the pathological tumour response. Furthermore the absolute SUV1-SUV2 difference was calculated (DSUV). If no residual metabolic activity was present on the pre-surgical PET-CT scan, the patient's tumour was classified as a metabolic complete responder, and the SUV was calculated in the same region of interest.

### Pathological tumour response

For each patient, the pathological tumour response was evaluated by determining the TRG (tumour regression grade), as proposed by *Mandard et al*. [7] All tumours were prospectively classified by an experienced pathologist (JLM) who was blinded to the PET data, as follows: TRG 1, complete tumour response; TRG2, residual cancer cells scattered through fibrosis; TRG 3, an increased number of residual cancer cells, with predominant fibrosis; TRG 4, residual cancer outgrowing fibrosis; and TRG 5, no regressive changes within the tumour. Based on the TRG, the tumours were grouped into responders (TRG 1 and 2) and non-responders (TRG 3-5). Furthermore, the pathological UICC classification (ypTN), including those with complete response (ypCR), was collected from the patients' specimen pathology report.

### Statistical analysis

Statistical analysis was performed using SPSS software (PASW Statistics 17.0.2). Comparison of the post-CRT SUVmax values vs. baseline was performed with the paired-samples t-test, whereas the independent-samples t-test was used to evaluate correlations between SUV, RI and DSUV values and patient's classification as responder or non-responder. Kruskal-Wallis test was employed to evaluate correlations between SUV, RI and DSUV values and the different TRG as well as the UICC ypStage. Differences were considered to be significant when the p-value was less than 0.05. The optimal cut-off value for therapy-related decrease in SUVmax was calculated by receiver-operating characteristic (ROC) analysis. Sensitivity, specificity, and positive and negative predictive values of 18F-FDG-PET-CT were calculated using standard formulas.

## Results

### Patients and tumours characteristic

37 (74%) males and 13 (26%) females were included. The age of the patients ranged between 36 and 80 years (mean 60). There were 35 (70%) patients with good to moderate and 15 (30%) with poor differentiated adenocarcinoma. 6 (12%) patients revealed mucinous components. In 31 (62%) patients capecitabine was used as chemotherapy combined with radiotherapy, in further 19 (38%) patients oxaliplatine was added according to hospital guidelines. Treatment plan was followed by all 50 patients. Tumour location ranged between 0 and 11 centimetres (cm) from the anal verge (mean 6 cm).

### Surgical data

Total mesorectal excision was performed in 48 patients (96%). There was 1 patient (2%) with high anterior resection (partial mesorectal excision) and another with local resection after complete response. 15 of 50 (30%) patients were submitted to abdomino-perineal resection (APR) surgery and 33 (66%) to low anterior resection (LAR). In 9 of 33 (27%) patients submitted to LAR, a permanent colostomy was left. In all other cases with LAR, protective ileostomy was indicated for three months. Timing between CRT and surgery ranged between 45-103 days (mean 59).

### Histopathological analysis

According to Mandard's criteria, the 50 patients treated with preoperative CRT and surgery were classified as TRG1 in 11 cases (22%), TRG2 in 9 (18%), TRG3 in 10 (20%), TRG4 in 12 (24%), and TRG5 in 8 (16%) (Table 1). According to the prognostic value of TRG score, they were classified into two groups: responders (TRG1-2; 20 patients [40%]) and non-responders (TRG3-5; 30 patients [60%]).

**Table 1:** Histopathological results: according to preoperative clinical UICC-Stage and tumor regression grade (TRG).

Stage n (%)	TRG1	TRG2	TRG3	TRG4	TRG 5	Total
**cStage II**	3 (6)	1 (2)	4 (8)	7 (14)	5 (10)	20 (40)

**cStage III**	8 (16)	8 (16)	6 (12)	5 (10)	3 (6)	30 (60)

**Total**	11 (22)	9 (18)	10 (20)	12 (24)	8 (16)	50 (100)

According to the UICC classification, i.e. TNM criteria, 10 patients (20%) were classified as ypStage 0 (ypCR), 15 patients (30%) as ypStage I, 11 patients (22%) as ypStage II, and another 14 patients (28%) as ypStage III (Table 2). Downstaging, no downstaging, and progression were found in 30 (60%), 19 (38%), and 1 (2%) patient, respectively.

**Table 2:** Histopathological results: according to UICC stage before and after CRT.

Stage n (%)	ypStage 0	ypStage I	ypStage II	ypStage III	Total
**cStage II**	3 (6)	10 (20)	6 (12)	1 (2)	20 (40)

**cStage III**	7 (14)	5 (10)	5 (10)	13 (26)	30 (60)

**Total**	10 (20)	15 (30)	11 (22)	14 (28)	50 (100)

### 18F-FDG-PET/CT findings

The pre-treatment (SUV1) values ranged from 4,85 to 34,56 (mean 13,67). After completion of neoadjuvant CRT, the glucose uptake (SUV2) values ranged from 2,36 to 15,85 (mean 5,73) (p < 0.001). DSUV and the regression index (RI) mean and SD values were 7,9 (6,2 SD) and 53,2 (23,3 SD), respectively. DSUV assumed negative value (SUV2 higher than SUV1) in 1 patient (2%). The median time between the end of CRT and the restaging PET and between PET and surgery was 42+6,3 and 17+10,7 days, respectively.

### 18F-FDG-PET/CT findings and pathological response

**Figure 3:**
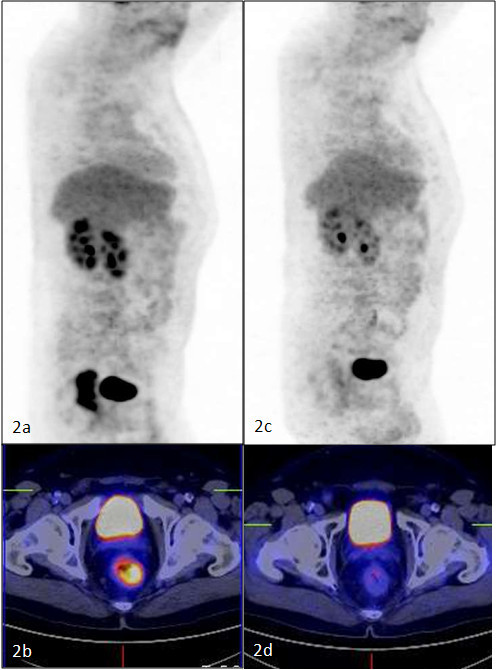
**Complete metabolic response to CRT**. 1A: Pre-CRT study - Intense rectal FDG uptake. 1B: Pre-CRT study - axial PET-CT images showing hypermetabolic rectal mass. 1C: Post-CRT study - Absence of rectal FDG uptake 1D: Axial PET-CT images showing rectal mass in CT images without FDG uptake.

The correlation between SUV1, SUV2, DSUV, and RI values resp. with the UICC Stage, and the TRG score was only statistically significant for SUV2 values (Table 3 and Figure 3). Results of ROC analysis for SUV1, SUV2, DSUV, and RI adjusted to the group of responders are resumed in Table 4. To elaborate the informative value of PET with respect to predictability of specific pathological response the cohort was divided into two dichotomous groups: ypCR vs. no-ypCR (Table 5) and TRG1-2 vs. TRG3-5 (Table 6). Stratifying the patients in responders and non-responders relating to regression grade (TRG1-2 vs. TRG3-5), the RI values were somewhat higher in the first group (59,65 +16,13 vs. 49,03+26,55, p = 0,116). The SUV1 and SUV2 values differed statistically significant between responders and non-responders (Table 6). DSUV was lower in the responder group than in the non-responder. ROC analysis found SUV2 to be the best predictor of response. Using SUV2 value of 4,24 as the cut-off threshold for defining response to therapy (AUC = 0.773, p < 0.001), it is possible to discriminate between responders and non-responders with a sensitivity of 70%, specificity of 76,6%, and PPV and NPV of 66,7%, and 79,3% respectively. The overall accuracy was 74% (Table 4).

**Table 3:** Tumor 18F-FDG uptake before (SUV1) and after neoadjuvant CRT (SUV2) according to UICC and Mandard's criteria (Kruskal-Wallis test).

	*n*	*SUV1*			*SUV2*			*DSUV*			*RI*		
		
		Mean	SD	p	Mean	SD	p	Mean	SD	p	Mean	SD	p
**UICC**				0,130			0,10			0,825			0,658
							
**0**	10	11,7	3,8		4,2	2,0		7,4	3,5		60,9	16,5	
							
**1**	15	15,7	7,1		6,4	2,7		9,3	8,1		53,1	23,1	
							
**2**	11	15,9	7,3		7,7	3,7		8,2	7,6		43,6	35,0	
							
**3**	14	10,9	4,0		4,4	1,2		6,4	3,8		55,5	14,6	

**TRG**				0,678			0,02			0,967			0,676
							
**1**	11	11,2	4,0		4,2	1,9		7,0	3,7		59,1	16,8	
							
**2**	9	12,2	3,8		4,5	1,5		7,6	3,4		60,3	16,1	
							
**3**	10	14,0	6,4		5,6	1,9		8,4	6,9		53,1	20,7	
							
**4**	12	15,1	7,1		6,4	2,7		8,7	8,1		51,1	24,1	
							
**5**	8	13,6	6,1		8,1	4,3		7,6	8,2		40,7	36,6	

**Table 4:** ROC analysis of 18F-FDG-PET findings for responders according to Mandard criteria (TRG1-2) and specifically to complete pathological response (ypCR).

Variable	End-point	Cutoff	AUC	p	Sens.%	Esp.%	PPV%	NPV%	Acc.%
**SUV1**	TRG1-2	**≤**10,07	0,618	0,160	45	70	50	65,6	60

	ypCR	**≤**10,14	0,615	0,243	45,5	74,4	33,3	82,9	68

**SUV2**	TRG1-2	**≤4,24**	**0,773**	**0,001**	**70**	**76,7**	**66,7**	**79,3**	**74**

	ypCR	**≤4,07**	**0,748**	**0,013**	**63**	**74,4**	**41,2**	**87,9**	**72**

**DSUV**	TRG1-2	**≥**8,90	0,545	0,593	45	70	50	65,6	60

	ypCR	**≥**9,735	0,508	0,935	36,4	71,8	26,7	80	64

**RI**	TRG1-2	**≥**62,75	0,623	0,14,3	55	70	55	70	64

	ypCR	**≥**69,67	0,585	0,393	45,5	74,4	33.3	82.8	68

**Table 5:** T test for ypCR vs. no ypCR

	Complete Pathologic Response	N	Mean	Std. Deviation	P value
SUV1	ypCR	11	11,2836	4,02432	0,149
		
	No ypCR	39	14,3403	6,54395	

**SUV2**	**ypCR**	**11**	**4,2427**	**1,98688**	**0,013**
		
	**No ypCR**	**39**	**6,1492**	**2,93618**	

RI	ypCR	11	59,1092	16,88429	0,354
		
	No ypCR	39	51,6421	24,82499	

DSUV	ypCR	11	7,0409	3,71728	0,594
		
	No ypCR	39	8,1910	6,78728	

**Table 6:** T test for responders (TRG 1-2) vs. non-responders (TRG 3-5)

	Response	N	Mean	Std. Deviation	P value
**SUV1**	**TRG 3-5**	**30**	**14,9740**	**7,08401**	**0,041**
		
	**TRG 1-2**	**20**	**11,7085**	**3,88062**	

**SUV2**	**TRG 3-5**	**30**	**6,6283**	**3,09551**	**0,001**
		
	**TRG 1-2**	**20**	**4,3820**	**1,77455**	

RI	TRG 3-5	30	49,0374	26,55346	0,116
		
	TRG 1-2	20	59,6560	16,13640	

DSUV	TRG 3-5	30	8,3457	7,54783	0,524
		
	TRG 1-2	20	7,3265	3,52060	

### 18F-FDG-PET/CT findings and ypCR

The SUV1 values were lower in the ypCR group than in the no-ypCR group (11,28 SD 4,02 vs.14,34 SD 6,54,(p = 0,149), on the contrary the RI values were higher comparing the same groups (59,10 SD 16,88 vs. 51,64 SD 24,82) (table 5). The SUV2 was significantly different in ypCR group vs. no-ypCR patients (Figure 3). ROC analysis identified a 4,07 (SUV2) as the cut-off value to predict ypCR (AUC = 0,748, p = 0.001); relative specificity and negative predictive value (NPV) were 74,4% and 87,9, respectively; whereas sensibility and positive predictive value (PPV) were 63% and 41,2, respectively; total accuracy was 72% (Table 4).

## Discussion

Positron emission tomography using fluoro-deoxy-glucose has demonstrated added value in the clinical management of patients with colorectal cancer [6]. This includes primary staging, detection of recurrence, prediction of individual prognosis, therapy response, and evaluation of treatment response as assessed in this investigation [8].

The interest in FDG-PET to assess tumour response to CRT began in the early 1990 s. Rectal cancer is a disease model of particular interest, not only for its high incidence, but also because an accurate and non-invasive method to evaluate response to preoperative CRT could lead to patients' selection for minimally invasive surgical approaches or even selection of candidates for additional chemotherapy and observation without any kind of surgery [2,3].

Experts at the Memorial Sloan-Kettering Cancer Center reported a pioneer prospective assessment of LARC response to preoperative CRT using FDG-PET in 2000 [9]. Today, literature is mixed in regard to the ability of 18-FDG-PET to predict response to neaodjuvant treatment in patients with rectal cancer. The majority of studies have reported post-treatment SUV to be lower than pre-treatment scans, but posttreatment SUV was not found to correlate with pCR. Furthermore, combining PET and CT with fusing of function and morphologic data has increased the sensitivity and specificity in restaging of various malignant tumours including LARC after CRT.

Recently, *de Geus-Oei *[8] analysed in an outstanding review the difficulty in comparing the outcome of different studies because of the use of several methods to analyse i.e. visual FDG-PET response, SUVmax, SUVmean, SUV ratio or even TLG (change in total lesion glycolysis) and that even at different intervals after CRT, varying from 12 days up to 7 weeks. It is interesting to note that all analysed papers found a significant relation of the investigated FDG-PET parameter to semiquantitative histological response [10-15]. Referring to response criteria, predictive values of FDG-PET response (negative predictive value) ranged between 83 to 100%; predictive values of FDG-PET non-response (positive predictive value) varied from 77 to 100%. The authors addressed that the more rigorous criteria of treatment response were defined the worse results were obtained [10-15].

Our results using SUVmax and performing the analysis 5 to 7 weeks after completion of CRT are in accordance with those found in literature [8]. We noticed a statistically significant difference between responders and non-responders according to Mandard's criteria for SUV 1 and SUV2 with a specificity of 76,6% and a PPV of 66,7%. Furthermore, SUV2 values were able to differentiate patients with complete pathologic response with a sensitivity of 63% and a specificity of 74,4% (PPV 41,2% and NPV 87,9%); This rather low sensitivity and specificity determined that PET-CT was only able to distinguish 7 patients with confirmed pCR from a total of 11 (4 cases were false negative). In addition to that, further 10 patients were false positive for pCR upon PET-CT.

While there are substantial data regarding the relationship between pCR and improved oncologic outcome, the prognostic significance of responders without pCR has not been extensively evaluated [16]. In our investigation, sampling the histopathological results according to the UICC (TNM) and Mandard's criteria appear to be in accordance with daily practice in hospitals. Whether Mandard's 1 and 2 classes belong both unequivocally to the responders is still a matter of discussion, on the contrary pCR seems to represent one of the most important prognostic factors leading to a more conservative surgical therapy and even to a wait and see non-resection policy in some series [2,3]. It should be also underlined that several publications have focussed on the prognostic value of metabolic response assessed by PET, independent of the final pathology report [17-19].

Time interval between the end of CRT and surgery and time interval between the end of CRT and posttreatment PET-CT scan are two variables not previously investigated that could affect the ability of PET scans to predict response to CRT. Cascini [20] and Janssen [21], have described the increased predictive value of FDG-PET when performed at an earlier and perhaps more relevant clinical stage, i.e.12 and 14 days (respectively) after CRT.

Our results are similar to those obtained using only PET for preoperative staging. Thus, the anatomical information obtained from the CT in a PET-CT scan does not seem to improve the detection rate of residual disease in our investigation. A drawback of post-CRT 18F-FDG PET is the radiation-induced inflammation that can accumulate approximately 25% of FDG update. On the other hand, direct effect of radiation may induce tumour cell dormancy ("stunning") that mimics response. Whether the different chemotherapeutical drugs used and combined with radiotherapy differentially affect the metabolism of the FDG at the tumour site is still unknown [22].

Our data are in accordance with literature that showed that PET-CT performed 5 to 7 weeks after completion of CRT can visualise functional tumour response in patients treated with neoadjuvant CRT. In contrast, metabolic imaging with FDG-PET is not able to predict pathologic complete response in LARC accurately.

## Conclusions

Our investigation identified PET-CT scan response as a complementary diagnostic and prognostic method in patients with locally advanced rectal cancer treated with neoadjuvant chemoradiotherapy. On the contrary, our results indicate that due to the rather low sensitivity and specificity, it does not seem possible to select patients upon metabolic imaging by means of 18F-FDG PET-CT to whom radical surgery after neoadjuvant CRT could be avoided.

## Competing interests

The authors declare that they have no competing interests.

## Authors' contributions

PP was responsible for overall planning, execution and interpretation of the study. ARF, RSS and MGR performed all nuclear studies, recorded and maintained PET data records. RCM, ISJ and JMC were responsible for surgical workout, including pathologic and oncologic data records. JAFO and JMLE contributed as senior members in planning and interpreting the study. All authors read and approved the manuscript.
